# The Effects of Risky Behaviors and Social Factors on the Frequency of Fraud Victimization Among Known Victims

**DOI:** 10.1093/geroni/igae111

**Published:** 2024-12-30

**Authors:** Marguerite DeLiema, Siyu Gao, Daniel Brannock, Lynn Langton

**Affiliations:** School of Social Work, University of Minnesota, Saint Paul, Minnesota, USA; School of Social Work, University of Minnesota, Saint Paul, Minnesota, USA; Center for Data Science, RTI International, Washington, District of Columbia, USA; Applied Justice Division, RTI International, Washington, District of Columbia, USA

**Keywords:** Loneliness, Lottery playing, Routine activity theory, Scam, Social isolation

## Abstract

**Background and Objectives:**

Routine activity theory (RAT) asserts that a suitable target’s exposure to a motivated offender in the absence of capable guardians increases their likelihood of crime victimization. We use these principles to assess the extent to which engaging in risky routine activities—for example, entering sweepstakes drawings, answering unknown calls—is associated with victimization frequency among older adult mass marketing fraud victims across five types of scams: investment fraud, sweepstakes and lottery fraud, romance and family/friend imposter scams, fake products and services, and charity scams. We also examine whether financial and social vulnerability characteristics (loneliness, preference for taking financial risks, financial fragility) are associated with victimization frequency in older adults.

**Research Design and Methods:**

A survey was administered to households that the U.S. Postal Inspection Service identified as having recently responded to one or more mail scam solicitations. Respondents answered questions on their behaviors, financial risk preferences, social and demographic characteristics, and number of past-year victimization experiences with 5 types of fraud.

**Results:**

As predicted based on RAT, routine activities that increase a target’s exposure to motivated offenders are positively associated with fraud victimization frequency, although more frequent online activity was negatively associated with victimization frequency contrary to hypotheses. Precarious financial and emotional states such as financial fragility and loneliness also were associated with greater victimization frequency, and more frequent social engagement and living with others (the presence of capable guardians) had no effect.

**Discussion and Implications:**

Target suitability factors such as loneliness, financial fragility, and risky financial preferences and behaviors are associated with a higher frequency of fraud victimization among older adults. Consumer education should include information on reducing risky behaviors that can increase fraud exposure. More frequent social engagement may not be protective. Older adults who are financially fragile and experiencing loneliness require more safeguards.

**Translational Significance:** Safeguarding older adults against fraud victimization requires recognizing and addressing needs related to financial insecurity and loneliness, as these characteristics make individuals more attractive targets to financial predators who use persuasive messages that promise to address these needs. Addressing other factors like reward-seeking behaviors and diminished sensitivity to financial risks may also help to reduce exposure to financial predators.

“Mass-marketing fraud” refers to any scam or fraud scheme that uses one or more mass-communication methods—such as the Internet, telephone, social media, text, or mail—to fraudulently solicit prospective victims (United States Department of Justice, 2023). Mass marketing fraud represents one form of financial exploitation that can harm older adults. Unlike elder financial abuse perpetrated by friends and family members (i.e., “trusted others”), fraud is generally perpetrated by predatory strangers and affects people of all ages (DeLiema, 2018). Fraud also involves an intentional act of deception whereby scammers convince targets to transfer funds under false pretenses that are either positive—a romantic relationship, an employment opportunity, a financial windfall—or negative—threat of arrest, stolen/lost data, kidnapped loved ones, extortion threats, and other harm.

Although adults of all ages are targeted by mass marketing fraud, victims aged 60 and older report median losses that are 2–3 times higher than young and middle-aged adults, ranging from $675 to $1750 per incident (FTC, 2023). In a systematic review and meta-analysis, Burnes and colleagues (2017) estimated that the annual prevalence of mass marketing fraud among older adults was 5.4% or about 1 in 18 older people. However, this is likely an underestimate because most people who experience fraud do not report it to authorities or to other sources (Raval, 2021; Schoepfer & Piquero, 2009).

Identifying risk factors for mass marketing fraud is challenging, as individuals who are most vulnerable may be underrepresented in general population surveys and in consumer complaint data. Moreover, most studies that examine risk factors treat fraud victimization as a binary outcome (yes/no), rather than an experience that can happen multiple times based on the risk factors that are present. To overcome these limitations, we survey a novel sample of U.S. adults who were identified by the U.S. Postal Inspection Service (USPIS) as having responded to one or more mass marketing frauds by mail. We call these individuals “known victims.”

## Study Purpose

The goal of this research is to assess what social and behavioral characteristics are associated with the self-reported frequency of mass marketing fraud victimization in a sample of older adult known victims after controlling for sociodemographic characteristics. Using routine activity theory (RAT; Cohen & Felson, 1979) as a theoretical lens, we predict that characteristics associated with target “suitability” (e.g., older age, loneliness, financial fragility, preference to take financial risks, and engagement in risky activities that increase fraud exposure), and the absence of capable guardians who can safeguard the target (e.g., low social engagement, living alone) are associated with self-reported fraud victimization frequency in the past year. Examining these risk factors in a sample of known fraud victims will facilitate a deeper understanding of the attributes that increase the risk of victimization in a vulnerable population. Additionally, by investigating modifiable behaviors and the social contexts that may increase/decrease fraud exposure and victimization risk, this study informs the development of tailored interventions and the placement of consumer education messages.

## Literature Review and Theoretical Background

### Routine Activity Theory

**Figure 1.**
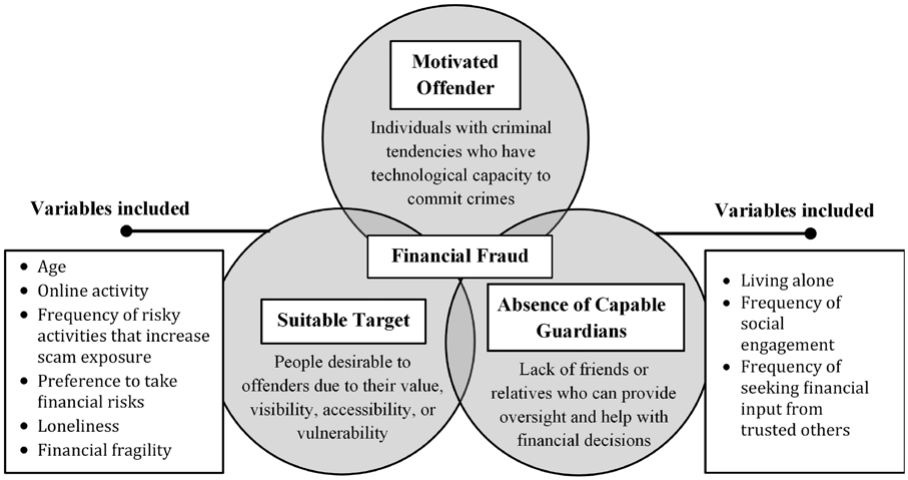
Routine activity theory as a conceptual framework for fraud victimization.

RAT was introduced by Cohen and Felson (1979) as a socioecological explanation for crime patterns in the United States. The theory emphasizes situational, contextual, and behavioral risk factors, that is, “routine activities,” beyond conventional demographic characteristics to explain the likelihood of victimization. According to RAT (Figure 1), criminal opportunities arise when suitable targets encounter motivated offenders in the absence of capable guardians, such as witnesses, security systems, and physical barriers. RAT was initially developed for criminological contexts such as sexual assault and property crimes but has also been used to describe risk factors for cybercrimes like identity theft (Burnes et al., 2020). There is a growing literature using RAT as a theoretical framework for elder fraud and financial abuse (Chan & Stum, 2020; DeLiema, 2018; Pratt et al., 2010; Reisig & Holtfreter, 2013; Xin et al., 2024). The present research investigates RAT-derived risk factors in a sample of independently identified fraud victims aged 60 or older who were administered a survey on past-year experiences with multiple forms of mass marketing fraud and other social and behavioral characteristics.

#### Suitable targets

According to RAT (Cohen & Felson, 1979), *suitable targets* are people or objects that are desirable to offenders because they are valuable, visible, accessible, and vulnerable. Older age is often posited as a factor that increases target suitability, although the literature is inconsistent (see Ross et al., 2014). Financial predators might perceive older adults as more valuable targets due to greater wealth accumulation than middle-aged and young adults (Gruijters et al., 2023) or more vulnerable due to lifestyle, cognitive, and social factors associated with aging (Lachs & Han, 2015). For example, changes in brain structure and the onset of mild cognitive impairment may impair financial decision-making, particularly in high-pressure persuasion contexts like scams (Han et al., 2016; Spreng et al., 2017). A recent study found a positive association between age and repeat fraud victimization in a large sample of fraud victims and their observed responses to scam solicitations over a 20-year period (DeLiema et al., 2024). Another study that used automated messaging to “scam-bait” financial predators online found that scammers preferred to engage with a fictitious persona programmed to communicate as an older widow relative to younger personas, suggesting that fraud criminals intentionally target older adults (Robinson & Edwards, 2024). Because aging is associated with factors that may increase target suitability based on perceived vulnerability and value, we hypothesize that:

***H1***: Age is positively associated with the frequency of self-reported fraud victimization.

According to RAT, routine activities that make a target more visible and/or accessible present opportunities for criminal victimization (Cohen & Felson, 1979). In the context of mass marketing fraud, behaviors that increase exposure to scam solicitations may increase risk. Several studies have found that remote investing and remote purchasing—for example, mail order and infomercial purchasing—are associated with fraud victimization (DeLiema et al., 2020; Holtfreter et al., 2008; Reisig & Holtfreter, 2013). In the present study, we examine respondents’ frequency of risky behaviors that may increase scam exposure, including opening and reading most mail (including advertisements), entering sweepstakes drawings to win gifts/prizes, answering unknown calls, and remaining on the phone with telemarketers.

Gainsbury and colleagues (2019) proposed that extensive online exposure increases the likelihood of victimization by amplifying offenders’ access to targets. Although the Internet is an essential tool for sharing information, connecting with others, and making financial transactions, it lacks regulatory supervision and enforcement (i.e., there is an absence of capable guardians). Research indicates that individuals who engage in greater online activity and willingly disclose personal information are more likely to experience online fraud (Mesch & Dodel, 2018; Van Wilsem, 2013). Prior research also showed that purchasing goods online increased the likelihood of being targeted by 377% (Pratt et al., 2010).

Although legitimate in many contexts, playing the lottery is a risk-taking financial activity that may appeal to opportunity-seekers who want to change their financial circumstances. Williams and colleagues (2017) propose that lottery and investment scams tend to target individuals who are motivated by financial gain, excitement, or a sense of challenge. Trahan et al. (2005) described a case study of a Ponzi scheme in which victims were promised substantial returns relative to their investments, similar to a lottery. In an experimental study investigating factors associated with susceptibility to phishing attacks, Rahman and colleagues (2023) found that the highest response rate to phishing messages occurred when participants were promised a Walmart gift card or an iPhone. Consumers who are drawn to opportunities that ask them to cover a small upfront cost for the opportunity to win big may be attractive targets for scams that follow a similar premise. We hypothesize that:

***H2***: Risky routine activities are positively associated with self-reported frequency of fraud victimization among older adults.

Similarly, we predict that those who are more open to taking chances with their money will be more likely to respond to scams that intentionally overestimate the potential benefits of an offer and minimize the risks. Individuals who are less sensitive to financial risk may be more suitable scam targets (Wood et al., 2023). We hypothesize that:

***H3***: Greater willingness to take financial risks is positively associated with self-reported frequency of fraud victimization among older adults.

DeLiema, Li, and Mottola (2023) found that *financial fragility*—having little to no access to emergency funds—was positively associated with losing money in opportunity-based scams such as fake jobs, government grants, advance fee loans, lotteries, and investments. Hall et al. (2022) similarly found that financial stressors were positively associated with financial exploitation vulnerability after controlling for demographic characteristics. Consumers seeking to improve their financial situation may be more open to money-making offers, making them more suitable targets for criminals peddling fraudulent investments, sweepstakes, and lotteries. Therefore, we hypothesize that:

***H4***: Financial fragility is positively associated with self-reported frequency of fraud victimization among older adults.

In addition to financial fragility, lacking companionship may increase target suitability. Individuals are often motivated to pursue or reestablish social connections in response to loneliness-related distress (Hawkley & Cacioppo, 2010) and some may address these feelings by forming digital connections (Cacioppo & Hawkley, 2009). In romance scams, investment frauds, and sweepstakes, prize, and lottery scams, financial predators use false promises to exploit targets’ emotional vulnerability—including feelings of loneliness—by building their self-esteem and making them feel special, important, and needed (Buchanan & Whitty, 2014; Olivier et al., 2016). Wong and Waite (2017) found a direct association between loneliness and financial exploitation, and DeLiema et al. (2023) demonstrated that individuals who felt lonely were more likely to engage in fraud solicitations. These studies suggest that lonely individuals may be more suitable targets. We hypothesize that:

***H5***: Loneliness is positively associated with self-reported frequency of fraud victimization among older adults.

#### Absence of capable guardians

RAT proposes that victimization is more likely to occur in the absence of capable guardians. Capable guardians may include friends or relatives who can provide financial oversight and assistance with financial decisions. According to DeLiema (2018), older adults who lack support from others to safeguard their assets and participate in financial decisions are more vulnerable to exploitation by financial predators. Moreover, scammers use undue influence to convince targets to keep their interactions and relationships a secret and to lie about the purpose of suspicious financial transactions to bankers and retailers (DeLiema et al., 2023). We hypothesize that:

***H6***: Social engagement is negatively associated with self-reported frequency of fraud victimization among older adults.

Individuals who engage trusted people in their financial affairs may be more protected from scams that aim to isolate them from capable guardians. We predict that:

***H7***: Seeking financial input from trusted others is negatively associated with self-reported frequency of fraud victimization among older adults.

#### Sociodemographic characteristics

Income, race, ethnicity, marital status, and education may affect the likelihood of being targeted by financial fraud (DeLiema & Witt, 2023; DeLiema et al., 2023). Although most sociodemographic factors are not promising modifiable targets for intervention, they may indicate which groups of people face higher risk and should receive enhanced consumer protection. Sociodemographic characteristics are included as controls in the analysis.

## Method

### Survey Sampling Frame—Identifying Fraud Victims

As part of their investigations into fraud, U.S. postal inspectors identify P.O. boxes or addresses that mass marketing fraud enterprises use to collect payments from victims and intercept envelopes headed to those addresses for victim protection. After a short investigation period, the USPIS returns the original payments to victims by feeding their envelopes through a machine that reads return addresses using optical character recognition. Fraud victims’ addresses in the present study were identified by the USPIS between July 2021 and January 2023 (17 months). The USPIS securely transferred the roster of victim addresses to the analyst team at RTI International. The list was uploaded to a secure server meeting the Federal Information Processing Standards moderate threshold. No names of victims or their household members were included. The study protocol and materials were approved by RTI International and University of Minnesota institutional review boards.

Analysts first reviewed the addresses for completeness and any errors that would result in mail being undeliverable. Of the 26,230 addresses on the initial roster, 8,625 were removed because they included no street address; 702 addresses were removed because they were missing key information such as street name or apartment number. With the invalid addresses removed, 16,903 valid addresses remained (64.4% of the total).

Next, analysts used Dedupe.io to identify duplicate addresses in the sample, meaning that the victim mailed money to scammers multiple times during the mail interception and investigation period. Only 2,857 unique addresses remained after the deduplication process (10.9% of the original list), indicating a high rate of repeat victimization. The USPIS provided the research team with a new list of 1,531 victim addresses in August 2023. After running Dedupe.io again with all available addresses and removing 307 duplicates, the final sampling frame included 4,081 unique addresses.

### Survey Administration

The survey (Supplementary Material, Section 1) was multimode with a web-based option and a mailed paper-and-pencil version. Households were first mailed a letter containing a unique link to the survey and a QR code. The letter explained that the purpose of the survey was to understand who is affected by mail scams and other types of fraud, provided survey access instructions, and informed respondents that participation was voluntary. Because the USPIS did not supply victims’ names, all survey mailings were addressed to “Postal Customer.”

To increase credibility, the invitation letter was co-branded with the USPIS, RTI International, and University of Minnesota logos. A $2 bill—visible through the clear window of the envelope—was sent with the initial invitation to increase response rates. A nonresponse strategy was implemented after 2 weeks that involved mailing (1) a postcard reminder including the participant’s unique survey link, (2) a paper copy of the survey, and (3) a final copy of the survey by Priority Mail to emphasize the urgency and value of the response. The final two mailings included prepaid return envelopes. All mailings were spaced approximately 2 weeks apart.

Survey collection ended on October 16, 2023 (~3 months), as survey responses had steadily dwindled. Ultimately, 934 completed surveys were collected: 15.5% from the web-based survey and 84.5% from returned paper-and-pencil surveys, resulting in an overall response rate of 23%. Survey data and codebooks are publicly available at the National Archive of Criminal Justice Data. Hypotheses are preregistered at https://aspredicted.org/9GB_SV4.

### Self-Reported Fraud Victimization Frequency

The dependent variable is constructed from items adapted from the Bureau of Justice Statistics’ Supplemental Fraud Survey (Langton et al., 2023; Morgan, 2021). Participants were asked if they paid, invested, or donated money in the past 12 months in response to five types of mass marketing fraud solicitations: (1) prizes, grants, and inheritance scams; (2) product and service scams (e.g., paying for products or services that were never received); (3) investment scams; (4) charity scams; and (5) family, friend, and romantic partner impersonation scams. For each item endorsed, participants were then asked, “How many times did this happen in the past 12 months?” Response options were 1 = “1 time,” 2 = “2–3 times,” 3 = “4–6 times,” 4 = “7–9 times,” 5 = “10 or more times,” and 6 = “Don’t remember/don’t know.” For those who selected a range, we assigned them the most conservative victimization frequency for that specific fraud type, for example, “2–3 times” = 2 incidents, “4–6 times” = 4 incidents, “7–9 times” = 7 incidents, and “10 or more times” = 10 incidents. Those who responded “don’t remember/don’t know” were conservatively assigned one incident. The five incident frequency variables were combined into a single measure representing the total self-reported fraud victimization frequency in the past year, ranging from 0 (no incidents; 34.6% of the sample) to 50 (all five forms of fraud at the highest incident frequency; 1.2%). Mean victimization frequency was 7.6 incidents with a standard deviation (*SD*) of 11.2. Because of the positive skew of the frequency distribution, the dependent variable was log-transformed to improve model fit. Model coefficients were exponentiated and 95% confidence intervals were calculated for ease of interpreting the results.

### Risky Routine Activities

To measure risky activities associated with greater target suitability, we used four items from the AARP Foundation National Fraud Victimization Study (Pak & Shadel, 2011). Participants rated the frequency of the following activities where 1 = almost every time, 2 = usually, 3 = occasionally, and 4 = almost never. Items were (1) Open and read most pieces of mail you receive, including advertisements; (2) Enter your name in sweepstakes drawings to win a prize or a gift; (3) Answer the phone when you do not recognize the caller; and (4) Hang up on telemarketers. The last item was reverse scored and responses were averaged (alpha = 0.39, mean = 2.40, *SD *= 0.63). Two additional items assessed whether respondents played lottery games: “In the past 30 days, have you spent money on…?” (1) instant-win or scratch-off tickets, and (2) lottery tickets (mean = 0.38, *SD = *.41).

### Financial Risk Preference

One item, adapted from the National Financial Capability Study (FINRA Investor Education Foundation, 2021), assessed financial risk preference: “How willing are you to take risks with your money for the potential to make more?” Responses ranged from “1” (very willing) to “5” (very unwilling). The item was reverse coded with higher scores indicating a greater willingness to take financial risks (mean = 2.57, *SD *= 1.31).

### Loneliness

Using the short version of the UCLA loneliness scale (Hughes et al., 2004), participants were asked to rate how often they felt (1) left out; (2) that they lack companionship, and (3) isolated from others on a scale of 1–3, where 1 = often, 2 = some of the time, and 3 = hardly ever or never. Responses were reverse scored and averaged with higher scores indicating greater loneliness (alpha = 0.81, mean = 1.74; *SD* = 0.62).

### Online Activity

Respondents were asked to rate the frequency of the following three behaviors, from “1” (every day) to “4” (almost never): “How often do you…?” (1) use the internet, either on a phone, tablet, or computer; (2) shop online; and (3) use social media, such as Twitter, Facebook, or Instagram. Responses were reverse coded and averaged. Higher scores indicate more frequent online activity (alpha = 0.72, mean = 1.70, *SD *= 0.81).

### Social Engagement

A two-item measure assessed social engagement frequency: “How often do you…?” (1) interact with friends, either in person or by talking on the phone; and (2) interact with family members, either in person or by talking on the phone. Responses ranged from “1” (every day) to “4” (almost never). Responses were reverse scored such that higher scores indicate greater social engagement (alpha = 0.80, mean = 2.48, *SD* = 0.91).

### Financial Input

One item assessed respondents’ frequency of seeking input on financial decisions from people they know and trust, from “1” (every day) to “4” (almost never). The item was reverse coded with higher scores indicating a greater frequency of seeking financial input from trusted others (mean = 1.48, *SD *= 0.71).

### Financial Fragility

Financial fragility is operationalized as an individual’s ability to cope with unexpected expenses. Using the measure of financial fragility reported in Lusardi et al. (2011), participants were asked about their confidence in being able to come up with $2,000 if an unexpected need arose next month. Those who responded that they were “certain they could not” or “probably could not” were coded as financially fragile (36.0% of respondents).

### Sociodemographic Characteristics

Respondents answered demographic questions last. Most characteristics were dichotomized for analysis, including gender (male/female), marital status (married/not married), educational attainment (high school or less/more than high school), household income (less than $50,000/$50,000 or more), race/ethnicity (non-Hispanic White/all other), and living arrangement (live alone/live with others). Survey response mode—online versus pencil and paper—was included as a covariate.

### Analytic Approach

We used multiple linear regression to examine the relationships between the risk factors and log-transformed frequency of fraud victimization in the past year. Missing responses ranged from 2% to 3% across the five fraud victimization items that comprised the dependent measure. Twenty-nine survey responses were excluded due to missing data on more than two of the five items. Because this study focuses on older adults, 83 respondents younger than age 60 were removed, resulting in a final sample of 823 respondents. Dichotomous missing variable flags were created for each independent variable where 1 = “response missing” and 0 = “response provided.” These missing flags were entered into the model as controls to retain the full sample. Simple mean imputation was used to address missingness in the continuous independent variables. To ensure the robustness of the results, we compared the model that imputed missing values and included missing flags to a model that used complete cases only and no imputation (*N* = 639; Supplementary Table 1). Results did not differ substantially, suggesting that the imputation method did not introduce significant bias.

We examined influential outliers and performed regressions with and without influential outliers removed (*n* = 4). Results did not differ, so these cases were retained in the model. Variance inflation factors ranged from 1.11 to 4.18, below the commonly used threshold of 5 (Sheather, 2009), indicating that multicollinearity is not a major concern. A bivariate correlation matrix is presented in Supplementary Table 2.

## Results

### Sample Characteristics

The average age of respondents was 77.7 years (*SD* = 8.9) and ranged from age 60 to 98. As shown in Table 1, 47% of respondents identified as male, 45% as female, less than 1% as transgender, less than 2% selected “none of these,” and 6% were missing. Approximately half (52%) were non-Hispanic White/Caucasian, 9% were Hispanic, 22% were Black, 6% were Asian/Asian Pacific Islander, 5% were Native American, 3% selected “other”, and 8% were missing. Approximately 26% were married, 34% were widowed, 21% were divorced or separated, 12% were never married, and 8% were missing or selected “prefer not to say.” Fifty-four percent reported a household income of less than $50,000, 18% reported an income of $50,000 or more, 18% preferred not to say or did not remember, and 9% were missing. Forty-six percent of respondents lived alone. Ten percent of respondents reported they had less than a high school education, 22% had a high school diploma or GED, 37% had some college or trade school, 23% had a bachelor’s degree or higher, and 8% were missing. Over one-third of respondents (36%) reported no fraud victimization, 17% reported just one scam type, 16% reported two scam types, 16% reported three scam types, 10% reported four, and nearly 5% reported that they experienced all five scam types.

**Table 1.** Sample Characteristics (*N* = 823)

Participant characteristics	*N*	%	Mean (*SD*)
Gender			
Male	387	47.0	
Female	369	44.8	
Transgender	3	.4	
None of these	13	1.6	
Missing	51	6.2	
Marital status			
Married/domestic partnership	213	25.9	
Widowed	277	33.7	
Divorced/separated	169	20.5	
Never married	97	11.8	
Missing	67	8.1	
Race (not mutually exclusive)			
White (non-Latino)	429	52.1	
Black	178	21.6	
Hispanic/Latino	75	9.1	
Asian/Asian Pacific Islander	47	5.7	
Native American	37	4.5	
Other	27	3.3	
Missing	62	7.5	
Educational attainment			
None or less than a high school diploma	79	9.6	
High school graduate	184	22.4	
Some college/associate’s degree	304	36.9	
Bachelor’s degree or higher	189	23.0	
Missing	67	8.1	
Household Income			
Under $20,000	218	26.5	
$20,001–$50,000	230	27.9	
$50,001–$100,000	92	11.2	
$100,001 or more	55	6.7	
Prefer not to say or don’t remember	151	18.3	
Missing	77	9.4	
Living arrangement			
Live alone	376	45.7	
Live with others	367	44.6	
Missing	80	9.7	
Scam types (self-reported)			
Prize, sweepstakes, or grant	417	50.7	
Product/services	362	44.0	
Investment	264	32.1	
Charity	172	20.9	
Romance	102	12.4	
Age			77.7 (8.9)
1-year frequency of self-reported fraud (0–50)			7.6 (11.2)

*Note*: *SD* = standard deviation.

### Regression Results

#### Suitable targets

Table 2 presents the exponentiated model results. Contrary to H1, we find that the self-reported frequency of fraud victimization among older adult scam victims is not greater for those in their 70s and 80s/90s, on average, compared to those in their 60s (e^β^_age 70–79_ = 0.97, 95% CI = [0.77, 1.24], *p* = .833; e^β^_age 80+_* *= 1.06, 95% CI = [0.83, 1.35], *p* = .652).

**Table 2.** Regression Coefficients on the Effects of Target Characteristics and Behaviors on the Log-Transformed Self-Reported Frequency of Financial Fraud Victimization (*N* = 823; *R*^2^ = 0.23)

Independent variables		Coefficient (e^β^)	95% confidence		*p*-Value
			Lower bound	Upper bound	
Suitable target	Age (70–79)	0.97	0.77	1.24	.833
	Age (80+)	1.06	0.83	1.35	.652
	MI age	0.96	0.65	1.40	.814
	**Risky routine activities**	**1.44**	**1.26**	**1.65**	**<.001**
	*MI risky routine activities*	*0.50*	*0.24*	*1.02*	*.059*
	**Frequency of online activity**	**0.86**	**0.76**	**0.97**	**.012**
	*MI online activity*	*0.51*	*0.25*	*1.07*	*.076*
	**Lottery playing**	**1.50**	**1.23**	**1.82**	**<.001**
	MI lottery playing	0.80	0.44	1.44	.457
	**Financial risk preference**	**1.14**	**1.07**	**1.22**	**<.001**
	MI financial risk preference	1.01	0.65	1.58	.962
	**Loneliness**	**1.46**	**1.28**	**1.67**	**<.001**
	MI Loneliness	0.91	0.56	1.45	.680
	**Financially fragile**	**1.30**	**1.05**	**1.61**	**.015**
	Financial fragility-prefer not to say	1.09	0.84	1.43	.513
	Financial fragility-don’t know	1.08	0.79	1.49	.614
	MI financial fragility	1.22	0.79	1.89	.380
Presence of capable guardians	Living arrangement (Live with others)	1.02	0.82	1.26	.882
	MI living arrangement	0.93	0.62	1.41	.743
	Social engagement frequency	0.94	0.85	1.03	.190
	**MI social engagement**	**2.72**	**1.25**	**5.91**	**.012**
	Seek financial input	1.10	0.98	1.23	.118
	MI seek financial input	1.11	0.63	1.96	.710
Sociodemographic characteristics	Gender (Male)	0.91	0.76	1.08	.279
	MI Gender	0.94	0.49	1.81	.852
	Marital Status (Married)	1.00	0.78	1.28	.998
	MI Marital Status	1.35	0.80	2.28	.259
	Education (High school or less)	1.04	0.87	1.25	.637
	MI Education	0.90	0.56	1.44	.655
	Income (less than $50,000)	0.87	0.68	1.11	.275
	***Income-prefer not to say/don’t know***	**0.70**	**0.53**	**0.94**	**.019**
	*MI income*	*0.67*	*0.43*	*1.05*	*.080*
	*Race (non-Hispanic White)*	*0.85*	*0.72*	*1.00*	*.066*
	MI Race	0.86	0.51	1.46	.578
Survey mode	Online	0.99	0.77	1.27	.937

*Notes*: Significant variables are bolded. Marginally significant variables are italicized. All variables beginning with “MI” are missing indicator flags coded dichotomously. Participants who did not respond to the item in the survey, or the items within the composite measure (e.g., loneliness, social engagement) are coded as “1” on the MI indicator. Participants who provided a response to the item or composite measure are coded as “0” on the MI indicator.

We find partial support for H2. Risky routine activities—like entering sweepstakes drawings and answering calls from unknown people—are associated with a higher frequency of victimization incidents (e^β^ = 1.44, 95% CI = [1.26, 1.65], *p* < .001), as is purchasing lottery tickets and/or scratch-off tickets (e^β^ = 1.50, 95% CI = [1.23, 1.82], *p* < .001). However, there was a negative and statistically significant association between being more active online and the frequency of fraud victimization (e^β^ = 0.86, 95% CI = [0.76, 0.97], *p *= .012) which is counter to our assumptions.

As predicted by H3, greater willingness to take chances with money (financial risk preference) was significantly positively associated with fraud victimization frequency (e^β^ = 1.14, 95% CI = [1.07, 1.22], *p* < .001). Those who reported that they were financially fragile and would struggle to cover a $2,000 emergency expense self-reported 30% more victimization incidents relative to those who could cover an unexpected expense (e^β^ = 1.30, 95% CI = [1.05, 1.61], *p* = .015), supporting H4. As predicted in H5, loneliness was positively associated with fraud victimization frequency such that for every additional degree of loneliness, victimization frequency increased by 46% (e^β^ = 1.46, 95% CI = [1.28, 1.67], *p* < .001).

#### Presence of capable guardians

We did not find support for H6 or H7. Living with others was not significant (e^β^ = 1.02, 95% CI = [0.82, 1.26], *p* = .882), nor was the frequency of social engagement with friends and family members (e^β^ = .94, 95% CI = [0.85, 1.03], *p* = .190). Contrary to our prediction (H7), the frequency of seeking financial input from trusted others was not protective (e^β^ = 1.10, 95% CI = [0.98, 1.23], *p* = .118). In the smaller model without imputation cases with missing values removed (Supplementary Table 1), seeking financial input from others was statistically significant and associated with a *higher* frequency of fraud victimization (e^β^ = 1.14, 95% CI = [1.01, 1.29], *p* = .031). These three findings do not support the RAT-derived hypothesis that the presence of capable guardians reduces the frequency of fraud victimization.

#### Sociodemographic characteristics

Few sociodemographic characteristics were associated with self-reported frequency of fraud victimization. Gender, marital status, race/ethnicity, and education were not statistically significant, although race/ethnicity trended toward significance (e^β^_White_ = 0.85, 95% CI = [0.72, 1.00], *p* = .056) such that minoritized respondents reported a 15% higher frequency of fraud victimization relative to non-Hispanic White respondents. Those who chose not to disclose their household income in the survey reported 30% fewer incidents of fraud in the past year relative to those who reported incomes of $50,000 or more (e^β^_Income: prefer not to say_ = 0.70, 95% CI = [0.53, 0.94], *p* = .019). Missing income (item left blank) also trended toward significance (e^β^_Income: missing_ = 0.67, 95% CI = [0.43, 1.05], *p* = .080).

## Discussion

Guided by RAT (Cohen & Felson, 1979), this study examined the relationship between risky financial preferences, behaviors, and social and financial contexts on the frequency of self-reported fraud victimization in a sample of independently verified older adult fraud victims. Findings provide partial support for RAT as a conceptual framework for victimization. In the present study, we find that targets’ “routine activities,” such as entering sweepstakes drawings, purchasing lottery tickets, opening marketing mail, not hanging up on telemarketers, and answering unknown calls, constitute risky behaviors associated with greater target suitability and victimization frequency. Given that many scams are perpetrated over the telephone and through the mail (FTC, 2023), engaging in these activities likely increases exposure to motivated offenders in the absence of capable guardians. Our results extend findings by Reisig and Holtfreter (2013) who found that mail order and infomercial purchasing was associated with fraud victimization among adults aged 60 and older. Findings also support research by DeLiema et al. (2020) showing that more frequent stock trading and purchasing investments sold through unsolicited calls, emails, television advertisements, or “free lunch” seminars significantly differentiated investment fraud victims from traditional investors.

Another factor associated with target suitability—preference for taking financial risks—was associated with victimization frequency. Like the investment fraud victims described in DeLiema et al. (2020), this mindset indicates a willingness to take chances with money for the chance to earn more, and scammers exploit this mentality using *phantom fixation—*activating fantasies of wealth, romance, and/or prestige—as a persuasion tactic. Financial risk-seeking individuals may be more easily persuaded by “get rich quick” schemes. Our findings align with an experimental study by Wood and colleagues (2023), which found that participants who rated a scam solicitation letter as having a high potential benefit and low risk were significantly more likely to indicate that they would respond.

Loneliness, another potential facet of target suitability, was significantly positively associated with victimization frequency as predicted. Older adults who experience loneliness may be more susceptible to fraud because they have unmet needs for social stimulation, emotional validation, and companionship. This finding corroborates numerous studies that have identified a link between vulnerability to fraud and loneliness and low social needs fulfillment (Alves & Wilson, 2008; DeLiema et al., 2023; James et al., 2014; Lichtenberg et al., 2016; Wen et al., 2022). Additional research is needed to explore how loneliness and other social factors affect the risk of victimization by specific types of fraud, including fear-based scams that use threats rather than promises of social connection.

In this sample of victims aged 60 and older, age was not significantly associated with self-reported frequency of fraud victimization. Our self-administered survey did not examine cognitive ability or decision-making capacity, and those with more severe impairment may not have responded to survey invitations. Future studies using samples of known mass marketing fraud victims should assess cognitive functioning to determine if cognitive impairment is a better proxy for target suitability than age alone.

Based on prior research (Pratt et al., 2010; Van Wilsem, 2013), we predicted that individuals who routinely use the internet and social media are more visible and accessible to fraud perpetrators. However, in this study focused on mail fraud victims aged 60+, more frequent online activity was *negatively* associated with fraud victimization frequency. This finding does not align with RAT, which posits that these behaviors would increase a suitable target’s exposure to motivated offenders (i.e., cybercriminals) in unsupervised environments. This discrepancy is likely due to our sample of participants who were identified as victims of mail-based scams rather than internet-facilitated scams. Further research is needed to understand the conditions under which online activity is protective or increases fraud risk.

As stated earlier, RAT proposes that victimization is more likely to occur in unsupervised settings where there is a lack of capable guardians who could prevent or deter crime. In general, we found less support for this component of RAT. We predicted that those who live with others, who are more socially engaged, and who seek financial input from people they know and trust would experience fewer scams, but these factors did not act as safeguards. It is possible that victims do not discuss solicitation offers with close friends and relatives (perhaps because they are instructed by scammers to keep them secret), or that they sometimes learn about fraudulent opportunities from trusted people in their social networks, such as in affinity-based multilevel marking scams and Ponzi schemes (Bosley & Knorr, 2018).

### Implications for Consumer Protection

Safeguarding older adults—and adults of all ages—requires recognizing and addressing unmet needs that make individuals more suitable targets. These may include financial insecurity and loneliness, as these characteristics make people more receptive to persuasive messages that promise to address these needs. One intervention approach to reduce loneliness is improving social engagement through meaningful activities. Sur and colleagues (2023) recently simulated the effects of a psychological well-being intervention and found that improving older adults’ well-being scores by even 10% caused significant reductions in scam susceptibility over time. Addressing other target suitability factors like reward-seeking and diminished sensitivity to financial risks is more challenging but may be addressed through financial literacy programs (Anderson, 2016; Drew & Cross, 2013), direct financial assistance, and help with money management.

This is the first study to show that purchasing lottery and/or instant-win scratch-off tickets is significantly associated with the frequency of financial fraud victimization. Lotteries offer fantasies about a better life and improved social position (Lutter et al., 2018), which is similar to the premise of many forms of fraud. Embedding consumer fraud education in settings where lottery tickets are sold, such as convenience and grocery stores, may help reach older adults who are persuaded by these messages. Education should include how to differentiate legitimate lotteries and sweepstakes from those that are fraudulent.

Other promising intervention targets are the risky behaviors that increase scam exposure, such as answering unknown calls and opening marketing mail. Older adults who engage in these behaviors need information on how to block unwanted calls or only allow inbound calls from known contacts. Education should include scripts for hanging up on telemarketers and throwing away junk mail. If these are meaningful routines for the older adult, behaviors may need to be replaced with more positive activities and connections with people (Deem & Lande, 2018).

### Limitations

The survey sample only included persons known to have experienced mail fraud. It did not have a comparison group of persons who had not responded to scams at all, or who were identified as victims of other forms of fraud. As such, respondents are not necessarily representative of fraud victims more broadly. Although this potentially impacts the generalizability of the findings, other studies have shown that many studies on fraud victimization are prone to social desirability bias, underreporting errors, false negative victim identification, and that they fail to include those who are most vulnerable (Beals et al., 2017; Parti & Tahir; 2023). This study addresses several of these shortcomings using a sample of mass marketing mail fraud victims who were objectively identified by the USPIS. However, the challenges of underreporting still exist since responses to the fraud victimization questions and number of incidents were self-reported. Factors such as guilt, shame, credulity, and poor recall may have reduced participants’ disclosure.

Without the respondents’ names, we cannot ascertain whether the individual who answered the survey is the same person within the household who paid money in response to the scam solicitations identified by the USPIS. We also lack information regarding the specific scams that USPIS investigated and how participants were initially solicited by the scammers, although we know they responded by mail.

Risky routine activities had a low alpha (0.39), driven by the item “frequency of hanging up on telemarketers.” It was not strongly correlated with the other items despite being conceptually related. Removing this item from the measure did not change the results. The individual items comprising this measure were also entered separately into a regression model (not shown), and all coefficients were in the same positive direction. Two were significantly associated with fraud frequency: not hanging up on telemarketers and entering sweepstakes drawings.

Many surveys were undeliverable because of missing address information, especially for apartment buildings. The characteristics of those who did not receive or respond to the survey may differ from those who responded. Non-responders may have been skeptical about participating, believing it to be another scam, or distrustful of the USPIS. Despite these factors, this study had a high response rate—23%—that exceeds response rates for most mail and online surveys.

## Conclusion

This is the first study to show the relationship between specific risky activities, such as buying lottery tickets and answering unknown phone calls, and the frequency of fraud victimization in a sample of older known victims. Although we found little support for the protective effects of frequent social interaction, seeking financial input from trusted others, and living with others (i.e., the presence of capable guardians), this study provides empirical support for the “suitable target” principle of RAT by demonstrating that loneliness, financial risk tolerance, financial fragility, and risky routine activities increase fraud risk. Interventions should seek to address targets’ social and financial needs and minimize risky activities that increase exposure to financial predators. Future research is needed to explore how to tailor fraud awareness education so that it is most effective with targeted populations, such as minority older adults.

## Supplementary Material

igae111_suppl_Supplementary_Material

## Data Availability

Data are being archived at the National Archive of Criminal Justice Data (NACJD), Inter-University Consortium of Political and Social Research at the University of Michigan. Hypotheses are preregistered and available at https://aspredicted.org/9GB_SV4.
