# Supplementary material for: The Effects of Risky Behaviors and Social Factors on the Frequency of Fraud Victimization Among Known Victims
Source: Innov Aging. 2024 Dec 30;9(2):igae111. doi: 10.1093/geroni/igae111 (PMC11809244; doi:10.1093/geroni/igae111)
Supplement: igae111_suppl_Supplementary_Material [file igae111_suppl_supplementary_material.docx]

***Innovation in Aging* Supplementary Material: DeLiema et al. The effects of risky behaviors and social factors on the frequency of fraud victimization among known victims**

**Section 1**. Survey instrument and codebook

## Instructions for print version of the survey:

*Please complete the survey by marking the appropriate responses. Use the enclosed prepaid envelope to return your completed survey to the research team at RTI International and University of Minnesota.*

## Instructions for electronic version of the survey:

*Please complete the survey by selecting the appropriate responses. You may skip questions that you prefer not to answer. The survey will take about 10 minutes to complete.*

## Prior-year experiences with mass marketing and other types of frauds

*The first section of questions asks you about experiences in the past 12 months in which someone may have convinced you to pay, invest, or donate money, by tricking or lying to you, hiding information, or promising you something that you never received.*

**puf_id** Unique identification code for individual respondents.

###### (Sequential six-digit ID code ranging from 100000 to 100933)

Variable type: Numeric

Field width: 6

**Survey_Source** Was collected by paper and pencil (Mail) or online (Web)?

1. Mail
2. Web

Variable type: Numeric

Field width: 6

**Group** To which group was the respondent assigned?

1. Control
2. Treatment Group 1
3. Treatment Group 2

Variable type: Numeric

Field width: 2

**State** State abbreviation for respondent.

AZ, AL, AR, etc. All 50 US states and the District of Colombia (DC) are included.

Variable type: String

Field width: 2

**Birth_Year** Birth year of the respondent, based on historical data obtained from scammers’ databases and linked to addresses of survey respondents.

#### (four digits ranging from 1919 to 1968)

9999 Historical data does not include DOB

Variable type: Numeric

Field width: 4

Missing: 88,9999

**Zip11_In_Historical_Data** This is a binary indicator for whether the address for that survey appears in the historical data. If 0 then there should be no event counts pre-2022.

1. No events prior to 2022
2. Yes

Variable type: Numeric

Field width: 1

**Multiple_people_with_zip11** The respondent’s address appears in the historical data, but is associated with multiple people (suggesting someone moved midway through the historical data). In that case, historical events are not counted since it is not possible to discern who was living at the address and filled out the survey. The ZIP11 refers to a specific address, so we only ever have one survey respondent per ZIP11.

1. No
2. Yes

88 Out of Universe

Variable type: Numeric

Field width: 1

Missing: 88

### Prize or grant fraud

**A1** In the past 12 months, have you paid money to receive a prize, grant, inheritance, lottery winning, or sum of money that you were told was yours?

1. Yes
2. No

Variable type: Numeric

Field width: 2

**A2** About how many times did this happen in the past 12 months?

1 1 time

2 2-3 times

3 4-6 times

4 7-9 times

5 10 or more times

1. Don’t remember/don’t know

88 Out of universe

99 No response

Variable type: Numeric

Field width: 2

Missing: 88, 99

**A3** Thinking about the most recent time, did you learn about the prize, grant, inheritance, or lottery from a letter or flyer you received in the mail?

1. Yes
2. No
3. Don’t remember/don’t know

88 Out of universe

99 No response

Variable type: Numeric

Field width: 2

Missing: 88, 99

### Product/Services

**A4** In the past 12 months, have you paid for any products or services that you NEVER received and NEVER got your money back for, or that turned out to be a SCAM? This might include buying something on the internet that you never got or paying for services that were not what you were promised.

- 1. Yes
  2. No

88 Out of universe

99 No response

Variable type: Numeric

Field width: 2

Missing: 88, 99

A5 About how many times did this happen in the past 12 months?

1 1 time

2 2-3 times

3 4-6 times

4 7-9 times

5 10 or more times

6 Don’t remember/don’t know

88 Out of universe

99 No response

Variable type: Numeric

Field width: 2

Missing: 6, 88, 99

A6 Thinking about the most recent time, did you learn about this/these products or services that turned out to be a scam from a letter or flyer you received in the mail?

1 Yes

2 No

3 Don’t remember/don’t know

88 Out of universe

99 No response

Variable type: Numeric

Field width: 2

Missing: 88, 99

### Investment fraud

**A7** In the past 12 months, have you invested money with a person or company that tricked you or lied to you about what you would receive, such as promising a guaranteed return on your investment or that you would not lose any money?

1. Yes
2. No

88 Out of universe

99 No response

Variable type: Numeric

Field width: 2

Missing: 88, 99

**A8** About how many times did this happen in the past 12 months?

1. 1 time
2. 2-3 times
3. 4-6 times
4. 7-9 times
5. 10 or more times
6. Don’t remember/don’t know

88 Out of universe

99 No response

Variable type: Numeric

Field width: 2

Missing: 88, 99

**A9** Thinking about the most recent time, did you learn about the investment opportunity from a letter or flyer you received in the mail?

1. Yes
2. No
3. Don’t remember/don’t know

88 Out of universe

99 No response

Variable type: Numeric

Field width: 2

Missing: 88, 99

### Charity fraud

**A10** In the past 12 months, have you donated money to a charity or a charitable cause that later turned out to be fake or that you later suspected was fake?

1. Yes
2. No
3. Don’t remember/don’t know

88 Out of universe

99 No response

Variable type: Numeric

Field width: 2

Missing: 88, 99

**A11** How many times did this happen in the past 12 months?

1. 1 time
2. 2-3 times
3. 4-6 times
4. 7-9 times
5. 10 or more times
6. Don’t remember/don’t know

88 Out of universe

99 No response

Variable type: Numeric

Field width: 2

Missing: 88, 99

**A12** Thinking about the most recent time, did you learn about the charity or a charitable cause from a letter or flyer you received in the mail?

1. Yes
2. No
3. Don’t remember/don’t know

88 Out of universe

99 No response

Variable type: Numeric

Field width: 2

Missing: 88, 99

### Romance and friend/family imposter scams

**A13** In the past 12 months, have you donated, sent, or otherwise given money to someone who PRETENDED to be a family member, friend, or someone interested in you romantically, but that person was not who they claimed to be?

1. Yes
2. No

88 Out of universe

99 No response

Variable type: Numeric

Field width: 2

Missing: 88, 99

**A14** How many times did this happen in the past 12 months?

1. 1 time
2. 2-3 times
3. 4-6 times
4. 7-9 times
5. 10 or more times
6. Don’t remember/don’t know

88 Out of universe

99 No response

Variable type: Numeric

Field width: 2

Missing: 6, 88, 99

**A15** Thinking about the most recent time, did that person send you a letter or letters by mail?

1 Yes

2 No

3 Don’t remember/don’t know

88 Out of universe

99 No response

Variable type: Numeric

Field width: 2

Missing: 88, 99

### Financial abuse

**A16** In the past 12 months, did someone close to you force or trick you into making a bad decision about your money?

1. Yes
2. No
3. Don’t remember/don’t know

88 Out of universe

99 No response

Variable type: Numeric

Field width: 2

Missing: 88, 99

**A17** How many times did this happen in the past 12 months?

1. 1 time
2. 2-3 times
3. 4-6 times
4. 7-9 times
5. 10 or more times
6. Don’t remember/don’t know

88 Out of universe

99 No response

Variable type: Numeric

Field width: 2

Missing: 6, 88, 99

**A18** In the past 12 months, did a friend, family member, or someone close to you make you give them money when you did not want to, or take your money, property, or possessions to use for themselves without your permission?

1 Yes

1. No

88 Out of universe

99 No response

Variable type: Numeric

Field width: 2

Missing: 88, 99

**A19** How many times did this happen in the past 12 months?

1 1 time

2 2-3 times

3 4-6 times

4 7-9 times

5 10 or more times

6 Don’t remember/don’t know

88 Out of universe

99 No response

Variable type: Numeric

Field width: 2

Missing: 88, 99

## Response to fraud

*[B1] This next section of questions asks you to think about any experiences you had in the past 12 months in which someone convinced you to pay, invest, or donate money, by tricking or lying to you, hiding information, or promising you something that you never received.*

*In response to these experiences did you…*

**B1a** Contact a law enforcement agency, such as the local police, a sheriff’s office, or a federal law enforcement agency to report the incident?

1 Yes

2 No

1. I don’t remember
2. Does not apply, this did not happen to me

88 Out of universe

99 No response

Variable type: Numeric

Field width: 2

Missing: 88, 99

**B1b** Contact a consumer agency, such as the Better Business Bureau, AARP, or the Federal Trade Commission?

1. Yes
2. No
3. I don’t remember
4. Does not apply, this did not happen to me

88 Out of universe

99 No response

Variable type: Numeric

Field width: 2

Missing: 88, 99

**B2_C** Would you want the Postal Inspection Service to alert you if they found out that you responded to scam mail?

- 1. Yes
  2. No
  3. Unsure

88 Out of universe

99 No response

Variable type: Numeric

Field width: 2

Missing: 88, 99

## Fraud victimization correlates

*The next questions are about certain aspects of your life.*

**C1** On an average weekday, how many letters do you receive in the mail? Please include letters you receive from businesses and other groups or organizations, as well as those from people you know.

1. None
2. 1-3 letters
3. 4-6 letters
4. 7-10 letters
5. 11-15 letters
6. More than 15 letters

88 Out of universe

99 No response

Variable type: Numeric

Field width: 2

Missing: 88, 99

**C2** Do you own or have access to a personal computer, such as a desktop or a laptop?

1. Yes
2. No

88 Out of universe

99 No response

Variable type: Numeric

Field width: 2

Missing: 88, 99

**C3** Do you own a smartphone or tablet?

1. Yes
2. No

88 Out of universe

99 No response

Variable type: Numeric

Field width: 2

Missing: 88, 99

*[C4] For the next series of questions, please respond to how frequently you do each of these activities.* ***How often do you….***

**C4a_1** [How often do you….] open and read most pieces of mail you receive, including advertisements?

- 1. Almost every time
  2. Usually
  3. Occasionally
  4. Almost never

99 No response

Variable type: Numeric

Field width: 2

Missing: 99

**C4a_2** *[How often do you….]* enter your name in sweepstakes drawings to win a prize or a gift?

1. Almost every time
2. Usually
3. Occasionally
4. Almost never

99 No response

Variable type: Numeric

Field width: 2

Missing: 99

**C4a_3** *[How often do you….]* answer the phone when you do not recognize the caller?

1. Almost every time
2. Usually
3. Occasionally
4. Almost never

99 No response

Variable type: Numeric

Field width: 2

Missing: 99

**C4a_4** *[How often do you….]* hang up on telemarketers?

1. Almost every time
2. Usually
3. Occasionally
4. Almost never

99 No response

Variable type: Numeric

Field width: 2

Missing: 99

**C4b_1** *[How often do you….]* the internet, either on a phone, tablet, or a computer?

1. Every day
2. Most days
3. Some days
4. Almost never

99 No response

Variable type: Numeric

Field width: 2

Missing: 99

**C4b_2** *[How often do you….]* shop online?

1. Every day
2. Most days
3. Some days
4. Almost never

99 No response

Variable type: Numeric

Field width: 2

Missing: 99

**C4b_3** *[How often do you….]* use social media, such as Twitter, Facebook, or Instagram?

1. Every day
2. Most days
3. Some days
4. Almost never

99 No response

Variable type: Numeric

Field width: 2

Missing: 99

**C4b_4** *[How often do you….]* interact with **friends**, either in person or by talking on the phone?

1. Every day
2. Most days
3. Some days
4. Almost never

99 No response

Variable type: Numeric

Field width: 2

Missing: 99

**C4b_5** *[How often do you….]* interact with **family members,** either in person or by talking on the phone?

1. Every day
2. Most days
3. Some days
4. Almost never

99 No response

Variable type: Numeric

Field width: 2

Missing: 99

**C4b_6** *[How often do you….]* volunteer for charitable organizations?

1. Every day
2. Most days
3. Some days
4. Almost never

99 No response

Variable type: Numeric

Field width: 2

Missing: 99

**C4b_7** *[How often do you….]* seek financial advice from people you know and trust?

1. Every day
2. Most days
3. Some days
4. Almost never

99 No response

Variable type: Numeric

Field width: 2

Missing: 99

### Loneliness

*[C5]* **How much of the time do you feel….**

**C5_1** *[How much of the time do you feel….]* left out?

1. Often
2. Some of the time
3. Hardly ever or never

99 No response

Variable type: Numeric

Field width: 2

Missing: 99

**C5_2** *[How much of the time do you feel….]* you lack companionship?

1. Often
2. Some of the time
3. Hardly ever or never

99 No response

Variable type: Numeric

Field width: 2

Missing: 99

**C5_3** *[How much of the time do you feel….]* isolated from others?

1. Often
2. Some of the time
3. Hardly ever or never

99 No response

Variable type: Numeric

Field width: 2

Missing: 99

**C5_4** *[How much of the time do you feel….]* bored in your day-to-day life?

1. Often
2. Some of the time
3. Hardly ever or never

99 No response

Variable type: Numeric

Field width: 2

Missing: 99

*[C6]* ***In the past 30 days, have you spent money on…***

**C6a** *[In the past 30 days, have you spent money on…]* instant win or scratch-off tickets

1 Yes

2 No

3 I don’t know

99 No response

Variable type: Numeric

Field width: 2

Missing: 99

**C6b** *[In the past 30 days, have you spent money on…]* other lottery tickets

1. Yes
2. No
3. I don’t know

99 No response

Variable type: Numeric

Field width: 2

Missing: 99

## Demographics

*[D] Next, we’re going to ask some questions about you and where you live.*

**D1** Do you currently describe yourself or identify as male, female, transgender, or some other way?

1. Male
2. Female
3. Transgender
4. Some other way
5. None of these

99 No response

Variable type: Numeric

Field width: 2

Missing: 99

**D2** Are you of Hispanic or Latino origin or descent?

1 Yes

2 No

99 No response

Variable type: Numeric

Field width: 2

Missing: 99

*[D3]* *What is your race? Please mark all that apply.*

**D3_1** White

1. Yes

77 Not selected

99 No response

Variable type: Numeric

Field width: 2

Missing: 77

**D3_2** Black or African American

1. Yes

77 Not selected

99 No response

Variable type: Numeric

Field width: 2

Missing: 77

**D3_3** American Indian or Alaska Native

1. Yes

77 Not selected

99 No response

Variable type: Numeric

Field width: 2

Missing: 77

**D3_4** Asian

1. Yes

77 Not selected

99 No response

Variable type: Numeric

Field width: 2

Missing: 77

**D3_5** Native Hawaiian or Other Pacific Islander

1. Yes

77 Not selected

99 No response

Variable type: Numeric

Field width: 2

Missing: 77

**D3_6** Other Race - please specify

1. Yes

77 Not selected

99 No response

Variable type: Numeric

Field width: 2

Missing: 77

**D4** What is your age? __________________________

## (two digits ranging from 20 to 98)

*[Note: Dated were collected in 2023. Users may calculate year of birth by subtracting age from 2023.]*

99 No response

Variable type: Numeric

Field width: 2

Missing: 99

**D5** What is your marital status?

1. Married / domestic partnership
2. Widowed
3. Divorced
4. Separated
5. Never married

99 No response

Variable type: Numeric

Field width: 2

Missing: 99

### Living arrangements

**D6** Which best describes your current living arrangement? (Check all that apply)

**D6_1** Live alone

1 Yes

77 Not selected

99 No response

Variable type: Numeric

Field width: 2

Missing: 77

**D6_2** Live with a spouse/partner

1 Yes

77 Not selected

99 No response

Variable type: Numeric

Field width: 2

Missing: 77

**D6_3** Live with dependent children or adult children

1 Yes

77 Not selected

99 No response

Variable type: Numeric

Field width: 2

Missing: 77

**D6_4** Live with extended family, such as siblings or grandchildren

1 Yes

77 Not selected

99 No response

Variable type: Numeric

Field width: 2

Missing: 77

**D6_5** Live with roommates

1 Yes

77 Not selected

99 No response

Variable type: Numeric

Field width: 2

Missing: 77

### Household composition

**D7** Including yourself, how many people age 18 or older currently live in this household? Include yourself, all family members, roommates, and boarders age 18 or older. _______

## (two digits ranging from 1* to 76**)

88 Out of universe

99 No response

Variable type: Numeric

Field width: 2

Missing: 88, 99

**On mail-in surveys, a few respondents entered “0”, not counting themselves. All zero values were recoded to “1” to account for the respondent.*

***Larger numbers are assumed to be individuals living in group home settings.*

**D7recode** Including yourself, how many people age 18 or older currently live in this household? Include yourself, all family members, roommates, and boarders age 18 or older.

Recoded to ordinal variable.

# (one digit ranging from 1 to 5)

1. One
2. Two
3. Three
4. Four
5. Five or more

88 Out of universe

99 No response

Variable type: Numeric

Field width: 2

Missing: 88, 99

**D8** How many children 0-17 currently live in this household? Please include small children and infants. _______

## (two digits ranging from 1 to 10)

88 Out of universe

99 No response

Variable type: Numeric

Field width: 2

Missing: 88, 99

**D9** Do you rent or own the place where you’re living?

1. Rent
2. Own
3. Other

99 No response

Variable type: Numeric

Field width: 2

Missing: 88, 99

### Educational attainment

**D10** What is the highest degree or level of school you have completed? If currently enrolled, highest degree received.

1. No high school diploma or equivalent
2. High school graduate, diploma or GED
3. Some college credit, no degree
4. Trade/technical/vocational training
5. Associate degree
6. Bachelor’s degree
7. Master’s degree
8. Professional degree
9. Doctorate degree

99 No response

Variable type: Numeric

Field width: 2

Missing: 99

### Income

**D11** Which category best fits the approximate TOTAL income of all members of your HOUSEHOLD during the past 12 months? Include money from jobs or other earnings, pensions, interest, rent, Social Security payments, and so on.

1. Under $20,000
2. $20,001 to $50,000
3. $50,001 to $100,000
4. $100,001 to $150,000
5. $151,000 to $200,000
6. $200,001 or more
7. Don’t remember
8. Prefer not to say

99 No response

Variable type: Numeric

Field width: 2

Missing: 99

**D12** How confident are you that you could come up with $2,000 if an unexpected need arose within the next month?

1. I am certain I could come up with the full $2,000
2. I could probably come up with $2,000
3. I could probably NOT come up with $2,000
4. I am certain I could NOT come up with $2,000
5. Don't know
6. Prefer not to say

88 Out of universe

99 No response

Variable type: Numeric

Field width: 2

Missing: 99

**D13** How willing are you to take risks with your money for the possibility to make more?

1. Very willing
2. Somewhat willing
3. Neither willing nor unwilling
4. Somewhat unwilling
5. Very unwilling

99 No response

Variable type: Numeric

Field width: 2

Missing: 99

**Section 2:**

Supplementary Table 1: Regression coefficients on the effects of fraud victim characteristics and behaviors on log-transformed self-reported fraud victimization; no imputation or missing flags (N=639; *R*^2^= 0.24)

| Independent Variables | | Exponential Coefficient  ($e^{\beta}$) | Confidence level (95%) lower limit | Confidence level (95%) upper limit | p-value |
| --- | --- | --- | --- | --- | --- |
| **Suitable Target** | Age (70-79) | 1.07 | 0.84 | 1.35 | 0.586 |
|  | Age (80+) | 1.08 | 0.85 | 1.37 | 0.554 |
|  | **Risky routine activities** | **1.40** | **1.20** | **1.63** | **<.001** |
|  | **Online activity** | **0.84** | **0.74** | **0.95** | **0.007** |
|  | **Lottery player** | **1.56** | **1.26** | **1.93** | **<.001** |
|  | **Financial risk preference** | **1.15** | **1.07** | **1.24** | **<.001** |
|  | **Loneliness** | **1.43** | **1.24** | **1.66** | **<.001** |
|  | Financially fragile | 1.31 | 1.05 | 1.63 | 0.017 |
|  | *Financial fragility-prefer not to say* | 0.98 | 0.74 | 1.29 | 0.868 |
|  | *Financial fragility-don’t know* | 1.03 | 0.72 | 1.47 | 0.862 |
| **Presence of Capable Guardians** | Living arrangement (Live with others) | 0.91 | 0.72 | 1.15 | 0.427 |
|  | Social engagement | 0.92 | 0.83 | 1.03 | 0.144 |
|  | **Seek financial input** | **1.14** | **1.01** | **1.29** | **0.031** |
| **Sociodemographic characteristics** | Gender (Male) | 0.89 | 0.74 | 1.07 | 0.227 |
|  | Marital Status (Married) | 1.03 | 0.79 | 1.35 | 0.816 |
|  | Education (High school or less) | 1.06 | 0.87 | 1.29 | 0.592 |
|  | Income (Less than $50,000) | 0.86 | 0.67 | 1.10 | 0.235 |
|  | *Income-prefer not to say/don't know* | 0.74 | 0.54 | 1.00 | *0.050* |
|  | **Race (non-Hispanic White)** | **0.82** | **0.69** | **0.98** | **0.032** |
| **Survey Mode** | Online | 1.08 | 0.83 | 1.40 | 0.581 |

Note: Statistically significant values are **bolded**. In this model, with no imputation or missing flags, race became significant, and the frequency of seeking financial input from known and trusted others became positively significant (a risk factor).

Supplementary Table 2: *Intercorrelations among variables included in the regression model*

|  |  | **1** | **2** | **3** | **4** | **5** | **6** | **7** | **8** | **9** | **10** | **11** | **12** | **13** | **14** | **15** | **16** | **17** | **18** |
| --- | --- | --- | --- | --- | --- | --- | --- | --- | --- | --- | --- | --- | --- | --- | --- | --- | --- | --- | --- |
| **1** | Financial fraud experiences | 1 | — | — | — | — | — | — | — | — | — | — | — | — | — | — | — |  |  |
| **2** | Risky routine activities | 0.267 | 1 | — | — | — | — | — | — | — | — | — | — | — | — | — | — |  |  |
| **3** | Online activity | -0.185 | -0.158 | 1 | — | — | — | — | — | — | — | — | — | — | — | — | — |  |  |
| **4** | Lottery player | 0.17 | 0.17 | -0.021 | 1 | — | — | — | — | — | — | — | — | — | — | — | — |  |  |
| **5** | Social engagement frequency | -0.127 | -0.022 | 0.35 | 0.072 | 1 | — | — | — | — | — | — | — | — | — | — | — |  |  |
| **6** | Financial risk preference | 0.231 | 0.226 | 0.079 | 0.169 | 0.001 | 1 | — | — | — | — | — | — | — | — | — | — |  |  |
| **7** | Loneliness | 0.204 | 0.148 | -0.163 | 0.049 | -0.163 | 0.052 | **1** | — | — | — | — | — | — | — | — | — |  |  |
| **8** | Gender (Male) | 0.038 | 0.091 | -0.068 | -0.03 | -0.188 | 0.222 | 0.009 | **1** | — | — | — | — | — | — | — | — |  |  |
| **9** | Age (70-79) | -0.02 | -0.036 | 0.086 | -0.001 | 0.026 | 0.013 | -0.028 | -0.05 | **1** | — | — | — | — | — | — | — |  |  |
| **10** | Age (80+) | 0.072 | 0.114 | -0.303 | -0.005 | -0.098 | 0.021 | -0.025 | 0.049 | -0.596 | **1** |  |  |  |  |  |  |  |  |
| **11** | Married | -0.091 | -0.082 | 0.196 | -0.002 | 0.112 | 0.058 | -0.237 | 0.094 | 0.079 | -0.046 | **1** | — | — | — | — | — |  |  |
| **12** | High school or less | 0.07 | 0.048 | -0.175 | 0.029 | -0.18 | -0.112 | 0.167 | -0.035 | -0.07 | -0.053 | -0.183 | **1** | — | — | — | — |  |  |
| **13** | Race (White) | -0.094 | -0.044 | 0.01 | -0.07 | 0.033 | 0.003 | -0.025 | 0.078 | 0.043 | 0.032 | 0.064 | -0.06 | **1** | — | — | — |  |  |
| **14** | Live with others | -0.078 | -0.002 | 0.164 | 0.055 | 0.182 | 0.023 | -0.25 | -0.039 | 0.079 | -0.063 | 0.635 | -0.087 | -0.01 | **1** | — | — | — | — |
| **15** | Seek input advice from others | 0.033 | 0.051 | 0.156 | 0.11 | 0.238 | 0.095 | -0.088 | -0.013 | 0.039 | -0.069 | 0.042 | -0.109 | -0.002 | 0.007 | **1** | — | — | — |
| **16** | Income less than $50K | 0.106 | 0.207 | -0.147 | 0.058 | -0.108 | -0.001 | 0.179 | 0.016 | -0.016 | 0.088 | -0.328 | 0.111 | 0.029 | -0.261 | 0.013 | **1** | — | — |
| **17** | Financial fragility | 0.102 | 0.098 | -0.149 | -0.001 | -0.054 | -0.072 | 0.163 | -0.135 | -0.056 | 0.01 | -0.215 | 0.155 | -0.01 | -0.097 | -0.071 | 0.273 | — | — |
| **18** | Survey mode (online) | -0.025 | -0.024 | 0.370 | -0.051 | 0.122 | 0.075 | -0.045 | 0.054 | 0.016 | -0.140 | 0.075 | -0.098 | -0.037 | 0.101 | 0.047 | -0.079 | -0.049 | **1** |
